# Supplementary figures and images for: Molecular dissection of laboratory contamination between two schistosome populations
Source: Parasit Vectors. 2024 Dec 22;17:528. doi: 10.1186/s13071-024-06588-9 (PMC11665219; doi:10.1186/s13071-024-06588-9)

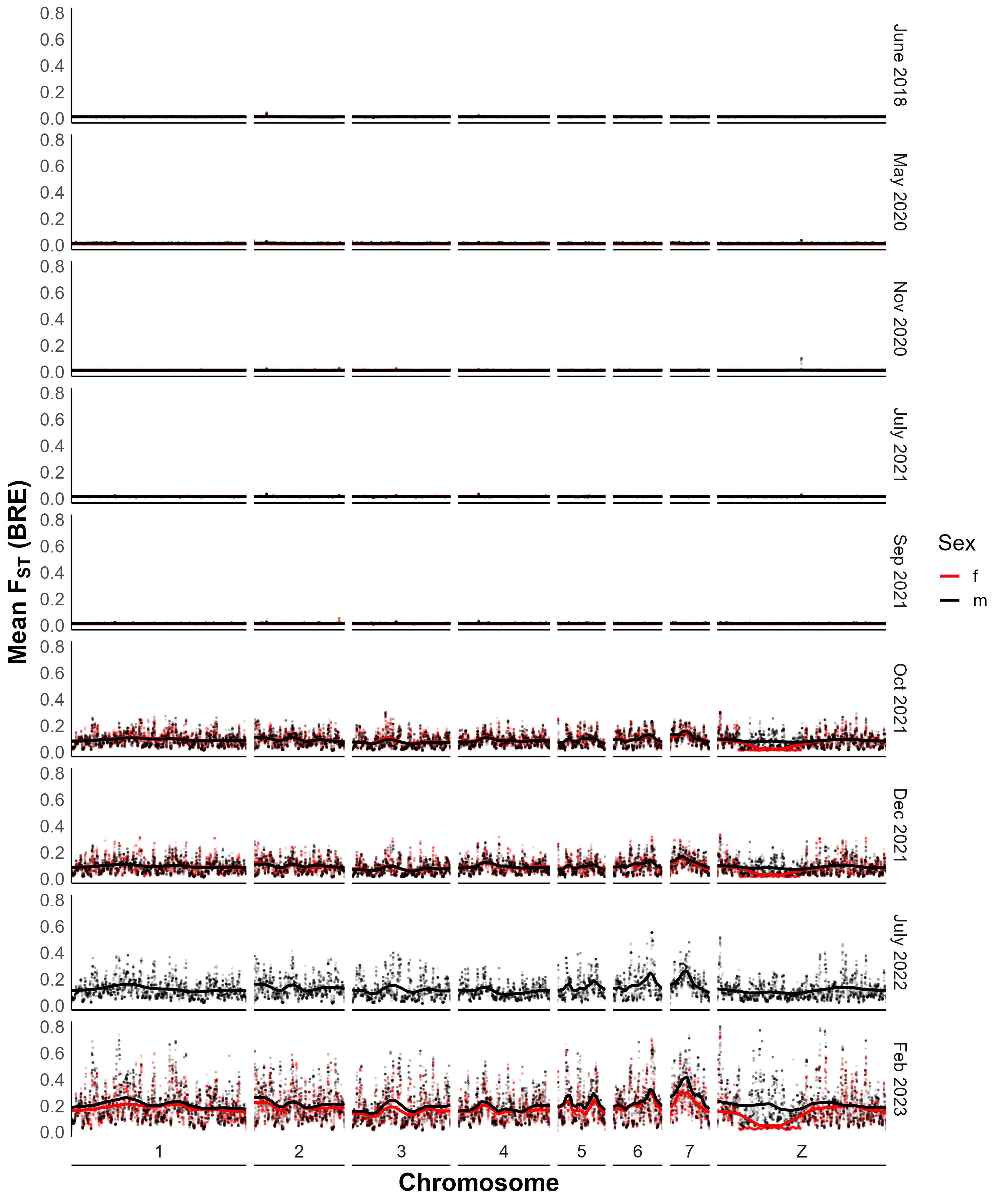

Supplement: Supplementary file 1 — Supplementary Material 1. Figure S1: Differentiation of SmBRE parasites between 2016 and all following time points. Dot plot showing smoothed average FST across the whole genome calculated in 20-kb windows. The solid lines indicate FST after smoothing with a local regression model as calculated by the locfit R package. [file 13071_2024_6588_MOESM1_ESM.jpg]

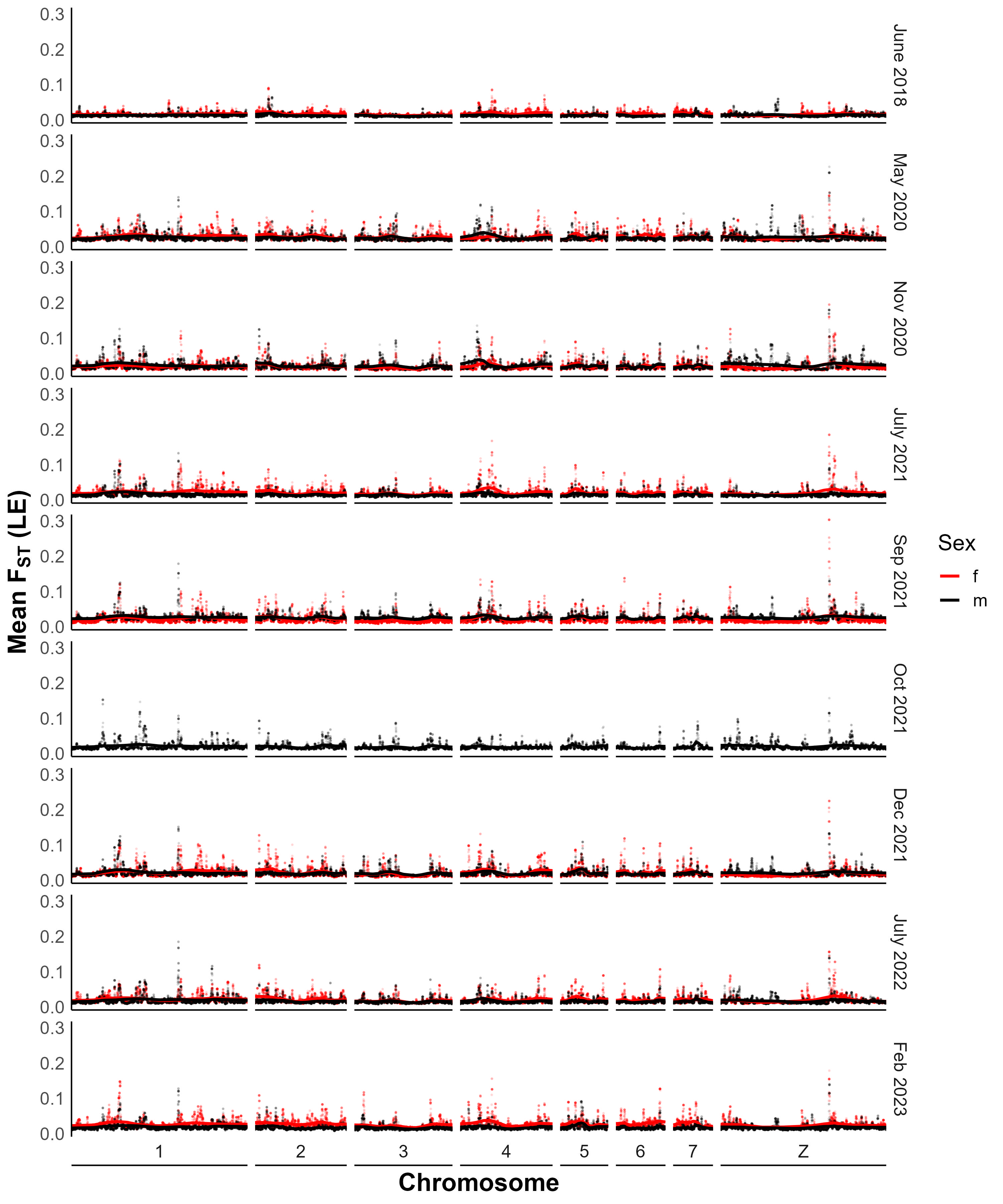

Supplement: Supplementary file 2 — Supplementary Material 2. Figure S2: Differentiation of SmLE parasites between 2016 and all following time points. Dot plot showing smoothed average FST across the whole genome calculated in 20-kb windows. The solid lines indicate FST after smoothing with a local regression model as calculated by the locfit R package. [file 13071_2024_6588_MOESM2_ESM.jpg]
